# Alteration of membrane complement regulators is associated with transporter status in patients on peritoneal dialysis

**DOI:** 10.1371/journal.pone.0177487

**Published:** 2017-05-19

**Authors:** Daniel Kitterer, Dagmar Biegger, Stephan Segerer, Niko Braun, M. Dominik Alscher, Joerg Latus

**Affiliations:** 1 Department of Internal Medicine, Division of Nephrology, Robert-Bosch-Hospital, Stuttgart, Germany; 2 Dr. Margarete Fischer–Bosch Institute of Clinical Pharmacology, University of Tuebingen, Stuttgart, Germany; 3 Division of Nephrology, Dialysis & Transplantation, Kantonsspital Aarau, Aarau, Switzerland; 4 Nephrology Center Stuttgart, Stuttgart, Germany; Postgraduate Medical Institute, INDIA

## Abstract

**Introduction:**

A growing body of evidence from animal models and cell culture studies indicate an important role of a local regulatory complement system (CS) in peritoneal injury during peritoneal dialysis (PD). We investigated the expression of the local regulatory CS (reflected by CD46,CD55,CD59) in the peritoneal tissue of patients with different membrane function characteristics.

**Patients and methods:**

Biopsies from the parietal peritoneum were taken from 24 patients on PD, 22 uremic patients prior to PD. PD patients were grouped according to the dialysate-to-plasma ratio of creatinine (D/P Cre) and ratio of dialysate glucose at 4 hours versus dialysate glucose at time zero (D/D0 glucose) into low or low-average peritoneal transport status (L/LA) and high-average or high-transport status (HA/H) groups. CD46, CD55, and CD59 RNA expression were analyzed by real-time polymerase chain reaction (RT-PCR). Further localization of membrane complement regulators (CRegs) and semiquantitatively analysis was done by immunohistochemistry (IHC).

**Results:**

CD46 and CD59 expression were similar in all groups. CD55 expression was significantly decreased in the HA/H group compared to the L/LA group and to uremic controls (p < 0.05 and p = 0.05, respectively). No statistically significant differences in CD46, CD55, and CD55 expression were detected when considering the history of peritonitis. There was no statistically significant correlation between PD duration and the expressions of CD46, CD55, and CD59. IHC revealed strong CD46, CD55, and CD59 expression in mesothelial cells. CD55 and CD59 were additionally detected in the vasculature. Using IHC, CD46 was lower in PD patients compared to uremic controls (p>0.05), but there was no difference between the L/LA compared to the H/HA group. Moreover IHC confirmed decreased expression of CD55 in the HA/H group compared to the L/LA group and uremic controls (p<0.0001 and p = 0.0001, respectively).

**Conclusion:**

CD55 expression is decreased in patients with fast transporter membrane function, whereas peritonitis and PD duration do not appear to alter CReg expression.

## Introduction

Loss of peritoneal membrane function is a major contributor to treatment failure in patients on peritoneal dialysis (PD) [1–3]. This may be due to either impaired solute clearance or ultrafiltration (UF) failure [3–5]. Non-specific morphological findings of the peritoneal membrane of patients with UF failure include thickening of the submesothelial layer due to extracellular matrix expansion (peritoneal fibrosis), neoangiogenesis, vasculopathy, and mesothelial cell alterations [6–8]. The long-time efficiency of PD is limited due to these chronic alterations of the peritoneal membrane, caused by various factors including osmotic stress, artificial catheter use, and peritonitis [9, 10]. Moreover, disturbances in aquaporin expression and water transport occur in long-term PD patients [11–14] and are a major factor for UF failure. UF is reduced by at least half in mice lacking the aquaporin-1 (AQP1) gene [15, 16], and loss of AQP1 (which is also present in capillaries of the peritoneal membrane) during long-term PD is strongly associated with UF failure [16–19]. This results in a reduction in the number of small pores and a relative reduction in the large-pore area over time [5, 20]. This might be the result of a shift from local inflammation at the beginning of PD [21, 22] to progressive fibrosis with increased vascular surface area due to long-term PD [5, 21, 23–25]. The function of the peritoneal membrane can be classified by measuring the rate at which solutes equilibrate between the dialysate and body plasma (dialysate-to-plasma ratio) [26]. Solute transport in PD is not only important for PD modalities [27], fast solute transport is also associated with a higher mortality risk and a trend to higher technique failure in PD patients [28, 29]. Transport characteristics and UF capacity of the peritoneal membrane vary among individuals and time on PD.

Activation of the complement system (CS) as a part of the “natural” immunity of the peritoneal cavity has recently been reported in PD patients [30–33]. Up to know, there is only less knowledge about the local CS and its regulation in the peritoneal membrane. There is growing evidence from animal models describing an important role of a local regulatory CS in the peritoneal cavity and association of disturbed membrane complement regulators (CRegs) with PM injury [34–37]. A recent cell culture study reported an abundantly expression of the CRegs CD46, CD55 and CD59 in human mesothelial cells [38]. Beside these Sei et al. showed a modified expression of the CReg CD55 in human mesothelial cells from PD patients with high peritoneal membrane solute transport [38].

To the best of our knowledge, there are no data regarding CReg expression in human peritoneal tissue. Consequently, we investigated the expression of CRegs, CD46, CD55 and CD59 in human peritoneal tissue and hypothesized that expression of local CRegs differ in patients with a faster dialysate-to-plasma ratio of creatinine compared to patients with a lower ratio.

## Patients and methods

### Patients and peritoneal biopsies

All peritoneal biopsies were obtained from the peritoneal biopsy registry at the Robert-Bosch-Hospital, Stuttgart, Germany. The collection of human peritoneal tissue, blood, and peritoneal dialysate for research purposes was approved by the local ethics committee (#322/2009BO1, Ethic committee, Eberhard-Karls University Tuebingen, Germany). All patients provided written informed consent concerning the scientific work-up of tissues taken during surgery.

Biopsies from the parietal peritoneum were taken from 24 patients on PD during hernia surgery (12 patients) or catheter explantation (12 patients). The uremic controls included 22 biopsies from uremic patients prior to PD initiation that were collected during catheter implantation. Peritoneal biopsies were washed in 0.9% saline solution, placed in RNAlater (Ambion, Woodlands, TX, USA) shortly after tissue excision, and stored at -80°C for subsequent RNA extraction. Real-time polymerase chain reaction (RT-PCR) was performed on these samples to analyze mRNA expression. PD patients were classified according to the dialysate-to-plasma ratio of creatinine (D/P Cre) and ratio of dialysate glucose at 4 hours versus dialysate glucose at time zero (D/D0 glucose) during a peritoneal equilibration test (PET) performed after 4 hours [26]. PET was performed at least 4 weeks after PD start. Patients were grouped according to D/P Cre ratio and D/D0 glucose into low or low-average peritoneal transport status (L/LA group) and high-average or high-transport status (HA/H group). For further analysis, we classified patients into groups with and without a history of PD-associated peritonitis. The patients’ baseline characteristics are shown in Table 1.

**Table 1:** Patient clinical data prior to and on PD.

Variable	Uremic controls	L/LA group	HA/H group	p
n	22	13	11	
Age (y; mean ± SD)	60.4 ± 14.0	58.2 ± 14.0	65.3 ± 14.7	n.s.
PD (duration in months)		12.0 (7.5–54.5)	22.0 (12.0–80.0)	n.s.
Peritonitis		12 in 403 months 1: 34	13 in 473 months 1: 36	n.s.
PDF				
Neutral		4/13	6/11	n.s.
Acidic		7/13	5/11	n.s.
Both		2/13	0/11	n.s.
Icodextrin		4/13	6/11	n.s.
Diabetes	5/22	5/13	2/11	n.s.
Smoker	4/22	5/13 1 N.D.	6/11 1 N.D.	<0.05^A^
Hypertension	18/22	9/13	9/11	n.s.
RRF (mL/24h)	1500 (1000–2000)	1000 (600–1500)	1200 (400–1900)	<0.05^B^
Laboratory findings				
Hb [g/dL ± SD (13–18)]	10.5 ± 1.3	11.6 ± 1.6	11.1 ± 1.8	<0.05^C^
Leukocytes [g/L ± SD (4.0–11.3)]	6.9 ± 2.1	6.8 ± 2.1	8.2 ± 3.8	n.s.
CRP [mg/dL ± SD (<0.1)]	0.9 ± 1.1	0.8 ± 0.8	2.5 ± 3.8	n.s.
Phosphate [mmol/L (0.68–1.68)]	1.8 ± 0.5	1.3 ± 0.5	1.4 ± 0.4	<0.01^D^ <0.05^E^
Calcium [mmol/L (1.90–2.70)]	2.2 ± 0.2	2.3 ± 0.3	2.3 ± 0.2	n.s.
PTH [pmol/L (1.1–7.3)]	33.7 ± 24.5	33.5 ± 18.4	30.0 ± 25.2	n.s.
Urea-N [mg/dL (10–25)]	129.3 ± 57.7	82.8 ± 21.6	81.5 ± 38.7	<0.01^F^ <0.05^G^
Creatinine [mg/dL (0.5–1.4)]	5.8 ± 2.1	6.3 ± 1.9	4.4 ± 1.8	<0.05^H^

CRP, C-reactive protein; Hb, hemoglobin; N.D., not determined; n.s. not significant, PD, peritoneal dialysis; PDF, peritoneal dialysis fluid; PTH, parathyroid hormone; RRF, residual renal function.

^A^ uremic controls vs. HA/H group;

^B^uremic controls vs. L/LA group;

^C^uremic controls vs. L/LA group;

^D^uremic controls vs. L/LA group;

^E^uremic controls vs. HA/H group;

^F^uremic controls vs. L/LA group;

^G^uremic controls vs. HA/H group;

^H^L/LA group vs. HA/H group; continuous data are expressed as means ± standard deviation (SD). Medians with interquartile ranges were used where distributions were not normal.

### RNA isolation

RNA was isolated from tissue immersed and frozen in RNAlater (Ambion). For RNA isolation mirVanaTM miRNA Isolation Kit (Ambion, Austin, TX, USA) according to the manufacturer’s protocol.

### Quality control

RNA was measured using a NanoDrop 2000c UV spectrophotometer (NanoDrop Technologies, Wilmington, DE, USA). RNA integrity was assessed using an Agilent 2100 Bioanalyzer (Agilent Technologies, Santa Clara, CA, USA).

### cDNA synthesis

400 ng total RNA from each specimen was reverse-transcribed using the High Capacity cDNA Reverse Transcription Kit (FisherScientific #10249814) for 10 min at 25°C, 120 min at 37°C, and 5 min at 85°C in a Veriti 96 well thermal cycler (Applied Biosystems). Resultant cDNA samples were diluted 5-fold.

### Quantitative RT-PCR

cDNA was performed employing TaqMan RT reagents according to the manufacture’s guidelines (Applied Biosystems). Relative quantification of mRNA expression was carried out using the standard curve method as previously described [39], normalized to 18SrRNA as an endogenous control, as it has been previously reported to be reliable across various conditions [40]. For *in vitro* studies, reverse transcription was performed, and the expression of candidate genes in samples was analyzed by the delta-delta Ct method [39]. Assay names are listed in Table 2.

**Table 2:** Characteristics of pre-developed TaqMan reagents.

Gene	Taqman Assay
18S ribosomal RNA	4319413E (Eukaryotic 18S rRNA Endogenous Control)
CD55	Hs00167090-m1
CD59	Hs00174141_m1
CD46	Hs00611257_m1

### Immunohistochemistry

Immunohistochemistry (IHC) was performed as previously described [41, 42]. Dewaxed and rehydrated tissue sections were incubated in Peroxidase Blocking Solution (S 2023; DAKO, Glostrup, Denmark). The antibodies and pretreatment conditions are listed in Table 3. We employed an Autostainer system (Autostainer Plus; DAKO) for IHC. The staining method used a dextran-coated peroxidase coupled polymer system (Dako REAL^™^ EnVision^™^ Detection Kit, Peroxidase/DAB+, Rabbit/Mouse, K 5007; DAKO), and hematoxylin counterstaining was performed.

**Table 3:** Characteristics of applied antibodies.

Antibody	Source	Dilution	Pretreatment
CD46	Santa Cruz Biotechnology (Santa Cruz, CA, USA), sc-9098 rabbit IgG	1:150	Steamer, pH 6
CD55	Abcam (Cambridge, UK), ab54595 mouse IgG1k	1:500	Steamer, pH 6
CD59	Abcam, ab9183 mouse IgG2b (Clone MEM-43/5)	1:200	Steamer, pH 6

#### Evaluation of CD46 immunoreactivity

Morphological CD46 immunoreactivity was described and semiquantitatively scored in mesothelial cells. Immunoreactivity of MCs was analyzed in high power fields (HPFs) as [absent, <3 cells/HPF, 4–10 cells/HPF, >10 cells/HPF (0,1,2,3)].

#### Evaluation of CD55 immunoreactivity

CD55 immunoreactivity was described and semiquantitatively scored in vessels and mesothelial cells. Immunoreactivity of mesothelial cells was analyzed as [absent, <3 cells/HPF, 4–10 cells/HPF, >10 cells/HPF (0,1,2,3)]. Five different representative HPFs on each slide were analyzed in a standardized manner. CD55 positive vessels were scored semiquantitatively as [absent, area of 1–10% positive vessels of 5 HPF, area of 11–50% of 5 HPF, and area >50% of 5 HPF (0,1,2,3)].

#### Evaluation of CD59 immunoreactivity

CD59 immunoreactivity was semiquantitatively scored in vessels and mesothelial cells. Immunoreactivity of mesothelial cells was analyzed as [absent, <3 cells/HPF, 4–10 cells/HPF, >10 cells/HPF (0,1,2,3)]. Five different representative HPFs on each slide were analyzed in a standardized manner. CD55 positive vessels were scored semiquantitatively as [absent, area of 1–10% positive vessels of 5 HPF, area of 11–50% of 5 HPF, and area >50% of 5 HPF (0,1,2,3)].

### Statistical analyses

All continuous variables were tested for normality using the Kolmogorov-Smirnov test. Comparisons between groups were made using the Mann-Whitney U test or Fisher’s exact test, as appropriate. Correlations were assessed by linear regression, and correlation coefficients were calculated by Pearson correlation analysis. All analyses were performed using GraphPad software (San Diego, CA, USA). Statistical results with a p-value <0.05 were considered significant. Continuous data are expressed as means ± standard deviation (SD). Medians with interquartile ranges were used where distributions were not normal. Error bars indicate SD.

## Results

### Patient characteristics

The clinical and laboratory data of 13 PD patients with low or low-average transport status (L/LA group), 11 PD patients with high-average or high-transport status (HA/H group), and 22 uremic control patients prior to PD are shown in Table 1. The mean ages were 58.2 ± 14.0 years in the L/LA group, 65.3 ± 14.7 years in the HA/H group, and 60.4 ± 14.0 in the uremic controls (p = 0.2, p = 0.6 and p = 0.4, respectively). The median PD durations were 12.0 (7.5–54.5) and 22.0 (12–80) in the L/LA and HA/H groups, respectively (p = 0.4).

### CD46, CD55, and CD59 mRNA expression in peritoneal biopsies of PD patients

**Fig 1:**
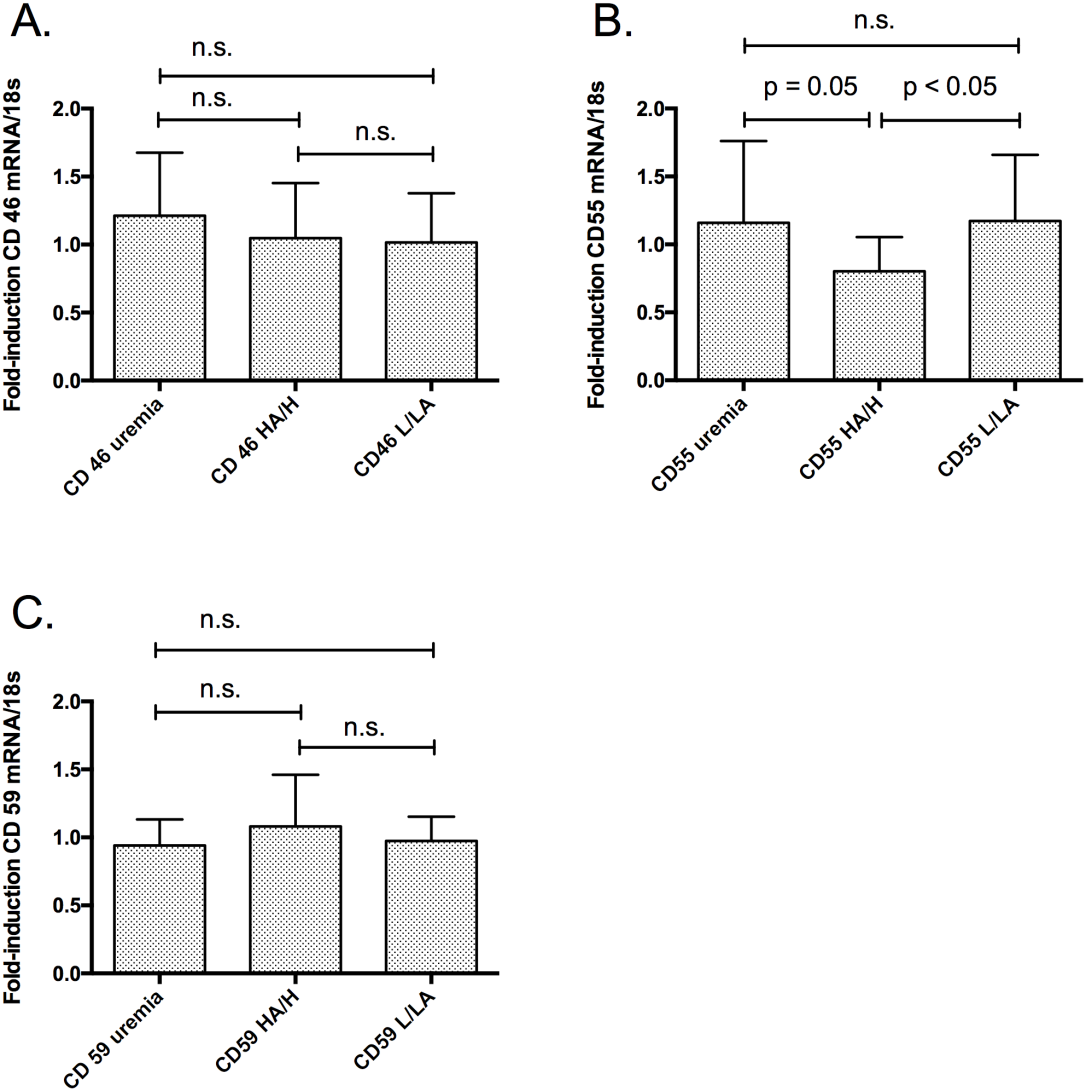
Expression of CD46, CD55 and CD59 in uremic controls (uremic patients at catheter implantation) and patients on peritoneal dialysis (PD). PD patients were grouped according the D/P ratio creatinine and the D/D0 glucose in patients with low or low average peritoneal transport status (L/LA group) and patients with high average or high transport status (HA/H group). RNA samples from uremic controls (n = 22), patients on PD in the L/LA-group (L/LA-group, n = 13), and patients on PD in the HA/H-group (HA/H-group, n = 11). CD46, CD55 and CD59 mRNA levels were analyzed by RT-PCR. The mean fold induction of CD46, CD55 and CD59 was normalized to the housekeeping gene 18SrRNA. (A.) There were no statistically significant differences in induction of CD46 RNA expression in PD patients compared to uremic controls and between the L/LA-group compared to the HA/H. (B.) The expression of CD55 was statistically significant decreased in the HA/H-group compared to the L/LA-group and to uremic controls (p < 0.05 and p = 0.05, respectively). (C.) There were no statistically significant differences in induction of CD59 RNA expression in PD patients compared to uremic controls and between patients in the L/LA-group compared to patients in the HA/H-group were detected.

Peritoneal biopsies from the three groups of patients were evaluated for CD46, CD55, and CD59 expression (Fig 1). CD46 was expressed in all three groups. There were no significant differences in expression of CD46 RNA in PD patients compared to uremic controls or between patients in the L/LA and HA/H groups (Fig 1A).

In contrast, CD55 expression was higher in the uremic control and L/LA group. No significant differences were detected between uremic controls and the L/LA group (p = 0.7). Interestingly, CD55 expression was statistically significant lower in the HA/H group compared to the L/LA and uremic control groups (p < 0.05 and p = 0.05, respectively) (Fig 1B).

CD59 expression was similar to that of CD46 in all groups. There were no significant differences in CD59 levels in PD patients compared to uremic controls, and there was no difference between the two PD groups (Fig 1C).

### CD46, CD55, and CD59 expression in peritoneal biopsies of patients depending on PD-associated peritonitis history

**Fig 2:**
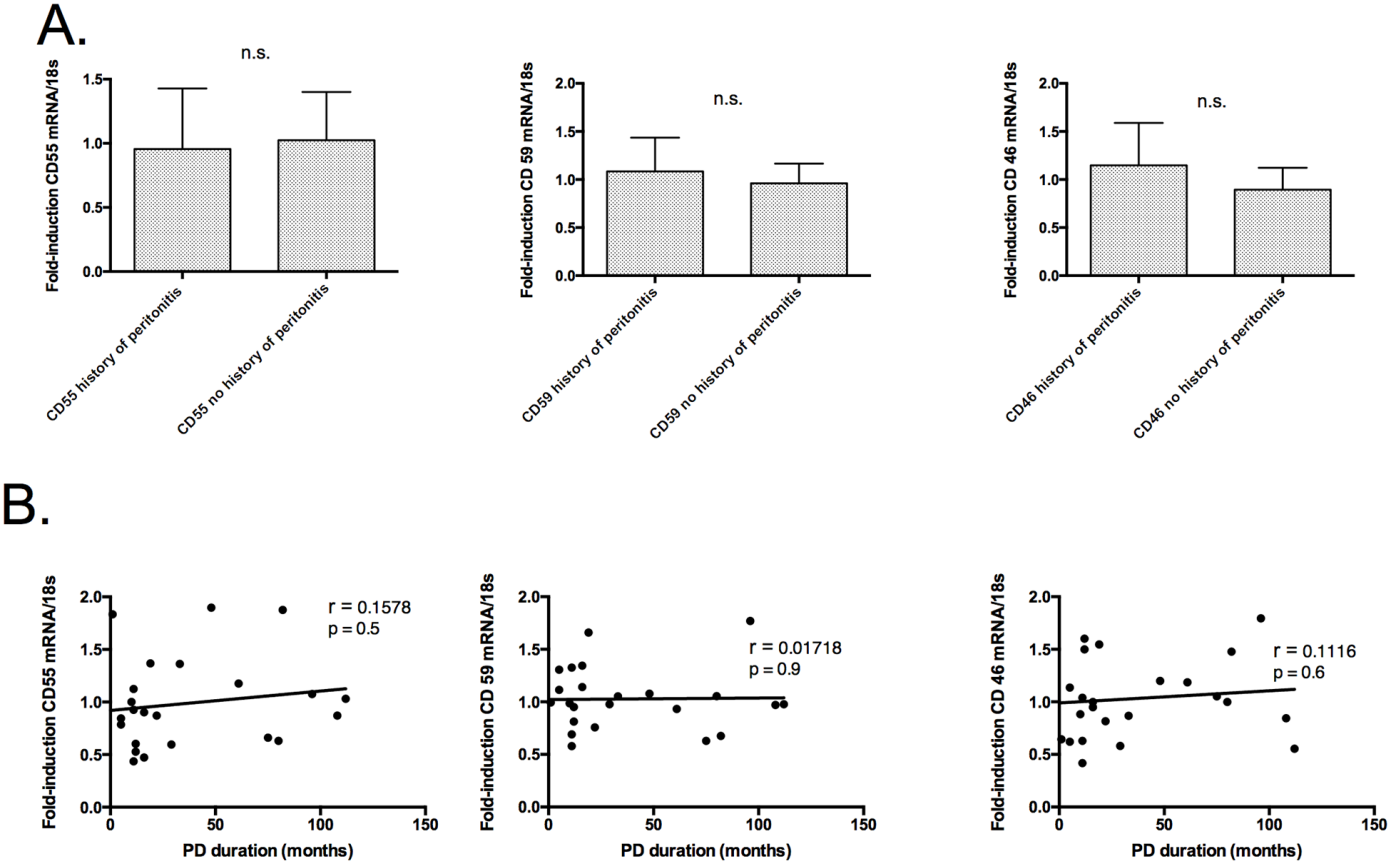
(A.) CD46, CD55, and CD59 expression in patients on PD based on their history of peritonitis. (B.) Correlation of PD duration and RNA expression of CD46, CD55, and CD59 in patients on PD. (A.) RNA samples from patients with history of peritonitis (n = 11) and patients without history of peritonitis (n = 13) were analyzed for CD46, CD55, and CD59 by RT-PCR. The mean fold inductions of CD46, CD55, and CD59 were normalized to the housekeeping gene 18SrRNA. There were no significant differences comparing patients with history of PD-associated peritonitis with patients without history of PD-associated peritonitis. (B.) There was no statistically significant correlation between PD duration and the expression of CD46, CD55, or CD55 in patients on PD.

Peritoneal biopsies were additionally evaluated for CD46, CD55, and CD59 expression depending on their history of PD-associated peritonitis (Fig 2A). Interestingly, we found no significant differences in the expressions of CD46, CD55, and CD55 in patients with history of PD-associated peritonitis compared to patients without a history.

### Correlation between PD duration and CD46, CD55, and CD59 expression in peritoneal biopsies of PD patients

There was no evidence of a correlation between PD duration and the expression of CD46, CD55, or CD55 (Fig 2B).

### Immunochemical analysis of CD46, CD55, and CD59

#### CD46 immunoreactivity

**Fig 3:**
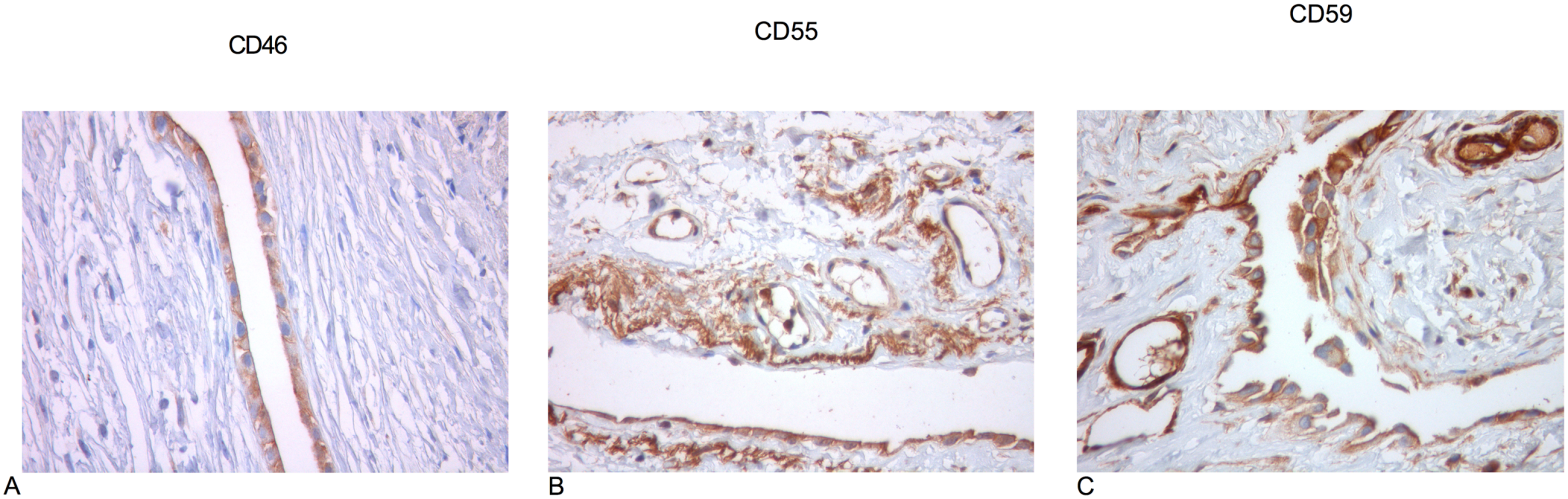
IHC of human peritoneal biopsies. (A.) CD46 staining was strong in mesothelial cells along the peritoneal surface in uremic controls and PD patients. (B.) CD55 was strongly positive in mesothelial cells along the peritoneal surface and in vessels in sub-peritoneal tissues in all groups. (C.) Intensive CD59 staining was observed in mesothelial cells in all groups. Vessels were positive for CD59 in uremic controls and PD patients. All images were taken at 400x.

CD46 staining was strongly present in mesothelial cells in all three groups. Interestingly, none of the samples showed vascular CD46 expression in the sub-peritoneal tissues (Fig 3A).

**Fig 4:**
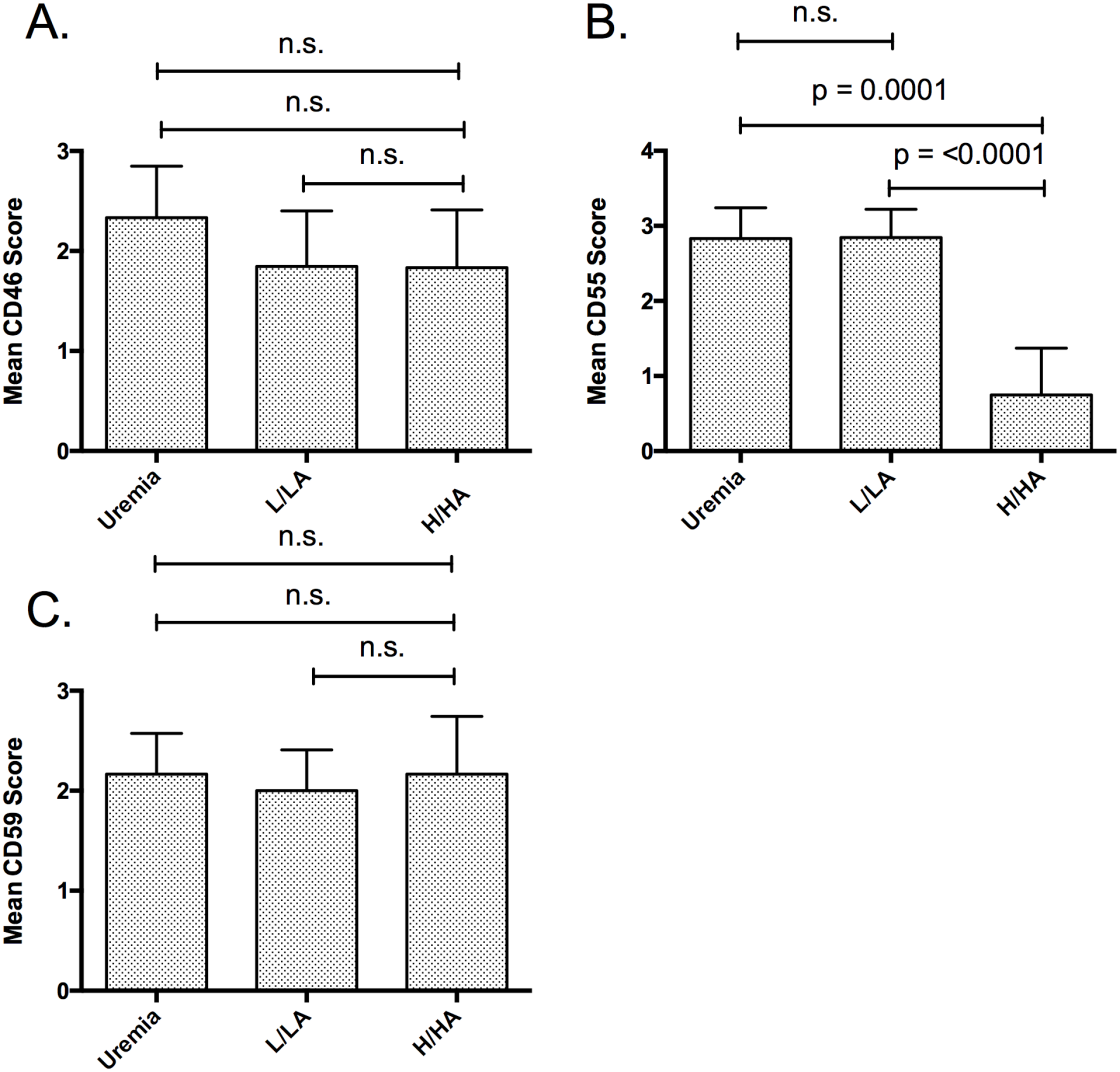
Expression of CD46, CD55 and CD59 using IHC of biopsies from uremic controls (uremic patients at catheter implantation) and patients on peritoneal dialysis (PD). **Expression was semiquantitative scored. PD patients were grouped according the D/P ratio creatinine and the D/D0 glucose in patients with low or low average peritoneal transport status (L/LA group) and patients with high average or high transport status (HA/H group)**. (A.) No statistically significant difference of CD46 staining between the groups was observed. (B.) CD55 staining was statistically significant decreased between the H/HA and the L/LA group and between the H/HA group and the uremic controls. No statistically significant differences between the L/LA group and the uremic controls could be observed. (C.) CD59 staining did not differ statistically significant between the groups.

Expression of CD46 was lower in PD patients (both the L/LA group and the H/HA group) compared to uremic controls, without reaching statistical significanceFurthermore, no statistically significant differences in the expression of CD46 in the L/LA group compared to the H/HA group was present (Fig 4A).

#### CD55 immunoreactivity

CD55 was strongly positive in mesothelial cells along the peritoneal surface, similar to CD46 expression in all three groups. Interestingly, we observed CD55 expression in occasional vessels in the sub-peritoneal tissues of uremic controls and PD patients (Fig 3B).

Expression of CD55 was equal in the L/LA group compared to uremic controls. The expression of C55 was statistically significant decreased in the H/HA compared to the L/LA group and compared to uremic controls (p = <0.0001 and p = 0.0001, respectively, Fig 4B).

#### CD59 immunoreactivity

Intense CD59 staining was observed in mesothelial cells, similar to the reactivity patterns of CD46 and CD55 in all groups. Vessels were also positive for CD59 in uremic controls and PD patients (Fig 3C).

No statistically significant differences was observed in the expression of CD59 between all three groups (Fig 4C).

## Discussion

Previous animal model studies reported that a loss of CReg function played an important role in peritoneal inflammation [35, 37], and the use of peritoneal dialysis fluid (PDF) decreased local CRegs in rats [36]. A recent cell culture study using primary cultures of mesothelial cells (HPMCs) from PD patients reported that expression of CD55 was associated with peritoneal membrane function (represented by a rapid equilibration of small solutes including creatinine and glucose) [38].

To the best of our knowledge, this is the first study to assess CReg expression in human peritoneal tissue from PD patients with different membrane function. The main findings of the study are as follows: *1)* Expression of the CRegs CD46 and CD59 were similar in the L/LA- and HA/H groups compared to uremic controls; *2)* CD55 expression was significantly decreased in patients with a D/P Cre ≥0.65 (HA/H group) compared to patients with a D/P Cre ≤0.64 (L/LA group) using both, RNA expression analysis and IHC staining; *3)* CD55 expression in the LA/L group was similar to uremic controls; *4)* no significant differences were found between the expressions of CD46, CD55, and CD55 when considering a history of PD-associated peritonitis; and *5)* there was no significant correlation between PD duration and the expression of CD46, CD55, and CD59.

The CS acts as a strong player of the innate immune system, and recent evidence suggests that it is important in modulating acquired immunity [43]. Moreover, complement activation and dysregulation play critical roles in various diseases [43, 44]. To date, little is known about CS activation and regulation in the peritoneal cavity of PD patients [31, 32]. In 1999, Barbano et al. reported that peritoneal mesothelial cells express CD59 and inhibits C5b-9-mediated cell lysis [33]. Moreover, CRegs play an important role in maintaining CS homeostasis [43]. Sei and colleagues reported that CD55 expression in HPMCs from PD patients was associated with D/P Cre. Additionally, HPMCs with lower CD55 expression were more susceptible to complement activation [38]. Our data confirmed these findings. IHC revealed strong staining for CD46, CD55, and CD59 in mesothelial cells of human peritoneal tissue. Moreover, CD55 and CD59 were also present in vessels.

CD46 and CD59 mRNA levels were similar in the L/LA and HA/H groups compared to uremic controls. In contrast to that, CD55 expression was significantly decreased in the HA/H group compared to the L/LA group and uremic controls using both, RNA expression analysis and IHC. Interestingly, we did not find significant differences between the expression levels of CRegs in patients regardless of their history of peritonitis.

It could be hypothesized that the PMs of patients with higher transport status are more vulnerable to complement activation than patients with lower transport status due to higher CD55 expression.

It is well known that a variety of stresses might be involved in membrane injury including “surgical injury”, artificial catheter, fluids, infections, osmolality [8, 45–49]. Previous reports suggest that the PD solutions lead to a down regulation of the CS in HPMCs [32] resulting in a potentially increased susceptibility to infections. It is well established that peritoneal solute transport rate represented by D/P Cre is increasing with time on PD [5]. But in some patients D/P Cre decreased after initial membrane function in the months after PD start [50, 51]. Interestingly, we found that the time on PD was not associated with significant differences in CReg mRNA levels. This indicates a primary association between solute transport rate and CReg expression status (perhaps on a genetic background), potentially resulting in an increased vulnerability of the membrane to complement activation in these patients.

Further studies are warranted in this field.

Our results confirmed previous cell culture studies that CD55 expression in human tissue is associated with the D/P Cre of PD patients [38]. PD duration and peritonitis do not seem to affect CReg expression patterns. Interestingly, human mesothelial cells and vasculature in the PM exhibited strong staining for CD55. It is well known that the integrity of mesothelial cells lining the peritoneal cavity decrease over time, whereas vessel surface increases [7, 23, 52]. Fast transporters achieve rapid and complete equilibration of small solutes due to a larger functional membrane surface area and higher membrane permeability [26, 53]. However, fast transporters quickly lose their osmotic gradient and achieve poor UF because dialysate glucose is rapidly absorbed from the peritoneal cavity [54, 55]. The expression of CD55 on vessels in this study indicates a vasculature disturbance in patients in the HA/H group.

Our study has several limitations that must be addressed. First, we included a relatively small sample size for expression analysis, which depends on the quality of mRNA collected from human biopsies. Second, we did not have access to peritoneal dialysate to analyze complement activation in this patient cohort. Third, we could not clarify the causes of altered CD55 expression in patients in the HA/H group. Hence, future studies that analyze longitudinal expression of CD55 and evaluation of membrane function in PD patients with different transport statuses over time should be performed.

In conclusion, our findings demonstrate that CD55 expression is decreased in patients with D/P Cre ≥0.65. Furthermore, the present study suggests that peritonitis and PD duration do not affect CReg expression patterns in human peritoneal tissue.
